# Copper foam supported *g-*C_3_N_4_-metal–organic framework bacteria biohybrid cathode catalyst for CO_2_ reduction in microbial electrosynthesis

**DOI:** 10.1038/s41598-023-49246-3

**Published:** 2023-12-20

**Authors:** Md Tabish Noori, Mansi, Shashank Sundriyal, Vishal Shrivastav, Balendu Sekhar Giri, Marcin Holdynski, Wojciech Nogala, Umesh K. Tiwari, Bhavana Gupta, Booki Min

**Affiliations:** 1 Department of Environmental Science and Engineering, Kyung Hee University, Yongin, South Korea; 2 CSIR-Central Scientific Instrument Organisation (CSIR-CSIO), Chandigarh, 160030 India; 3 Regional Center of Advanced Technologies and Materials, The Czech Advanced Technology and Research Institute (CATRIN), Palacký University Olomouc, Šlechtitel °u 27, Olomouc, 779 00 Czech Republic; 4 Institute of Physical Chemistry Polish Academy of Sciences, Kasprzaka 44/52, 01-224 Warszawa, Poland; 5 Sustainability Cluster, School of Advanced Engineering, UPES, Dehradun, Uttarakhand 248007 India

**Keywords:** Biotechnology, Chemistry, Energy science and technology, Engineering, Materials science, Nanoscience and technology

## Abstract

Microbial electrosynthesis (MES) presents a versatile approach for efficiently converting carbon dioxide (CO_2_) into valuable products. However, poor electron uptake by the microorganisms from the cathode severely limits the performance of MES. In this study, a graphitic carbon nitride (*g-*C_3_N_4_)-metal–organic framework (MOF) *i.e.* HKUST-1 composite was newly designed and synthesized as the cathode catalyst for MES operations. The physiochemical analysis such as X-ray diffraction, scanning electron microscopy (SEM), and X-ray fluorescence spectroscopy showed the successful synthesis of *g-*C_3_N_4_-HKUST-1, whereas electrochemical assessments revealed its enhanced kinetics for redox reactions. The *g-*C_3_N_4_-HKUST-1 composite displayed excellent biocompatibility to develop electroactive biohybrid catalyst for CO_2_ reduction. The MES with *g-*C_3_N_4_-HKUST-1 biohybrid demonstrated an excellent current uptake of 1.7 mA/cm^2^, which was noted higher as compared to the MES using *g-*C_3_N_4_ biohybrid (1.1 mA/cm^2^). Both the MESs could convert CO_2_ into acetic and isobutyric acid with a significantly higher yield of 0.46 g/L.d and 0.14 g/L.d respectively in MES with *g-*C_3_N_4_-HKUST-1 biohybrid and 0.27 g/L.d and 0.06 g/L.d, respectively in MES with *g-*C_3_N_4_ biohybrid. The findings of this study suggest that *g-*C_3_N_4_-HKUST-1 is a highly efficient catalytic material for biocathodes in MESs to significantly enhance the CO_2_ conversion.

## Introduction

There has been a significant increase in air pollution since many years due to an unchecked rise in carbon dioxide (CO_2_) emissions^1^. To date, significant effort has been made to reutilize CO_2_ on a variety of levels^2^. Thus, repurposing CO_2_ as other valuable chemicals is one of the many efforts and initiatives in this regard^3,4^. A method known as Carbon Capture and Utilisation (CCU) is now being used to capture CO_2_ and further transform it into several valuable commodities such as volatile fatty acids (VFAs) and their corresponding alcohols. This promising strategy will not only reduce atmospheric CO_2_ levels but will also address the difficulties of the current and upcoming energy crisis^5^. In this line, numerous methods, including thermochemical^6^, photocatalytic^7,8^, photoelectrochemical^9^, and electrochemical ^10–17^, and microbial electrochemical processes^18,19^, have been proposed to transform CO_2_ into value-added compounds. The electrochemical approach stands out as the best among all of these options because it is small in size and is simple to operate using electrode potentials^20^. The need for potential energy to drive the electrons can be met by using renewable energy sources including photovoltaics, wind, hydroelectricity, tidal, and geothermal energy, among others^21^. This electrochemical approach, nonetheless, is frequently constrained by inefficient catalysis, and inadequate product selectivity (able to produce mostly less expensive C1 organic compounds)^22^. Integration of microbes with electrochemical approaches can get over these restrictions and one such technology is called microbial electrosynthesis (MES)^23,24^.

An MES is usually fabricated with a pair of electrodes as an anode and cathode separated with a cation exchange membrane (CEM). A desired voltage is applied at the electrodes to drive the electrons generated at the anode via either water oxidation or organic matter oxidation to the cathode. At the cathode, electroautotrophic microorganisms employ these electrons to reduce CO_2_, leading to the production of various volatile fatty acids (VFAs) through a series of metabolic reactions facilitated by specific proteins and enzymes. During the metabolic process, microbes generate a central metabolite called Acetyl Co-A, which plays a pivotal role. It serves as a key intermediate in various biochemical pathways and is a precursor for the synthesis of many important molecules, including higher-chain fatty acids like butyric and valeric acids. Furthermore, microbial cathodes exhibit strong regenerative and resilient characteristics. This is due to the intact structure of the biofilm and the presence of suitable supporting materials (extracellular polymeric substances), which facilitate the continued growth of microorganisms within the biofilm. Thus, an MES can be operated for a longer time without the need for change in cathode catalysts^25,26^. This unique feature of self-sufficient biocatalysts provide MES an edge over conventional electrochemical processes where catalyst material degradation over time poses significant challenges. Nonetheless, a notable concern in MES is the inefficient electron transfer from the cathode to the microbes^27^. This significantly reduces the pool of available reducing equivalents for CO_2_ reduction in the microbes^28^. This challenge primarily stems from the inadequate interaction between microbes and the cathode, which is attributed to suboptimal material properties including limited active sites, low surface area, and poor conductivity^29,30^. Thus, the recent research in MESs has propelled the development of highly active cathode materials to improve the microbe-electration, resulting in high current responses^31,32^.

Porous materials in this context have demonstrated a strong affinity for microbes, facilitating the formation of robust conductive biofilms. A recent investigation employing porous Fe_x_MnO_y_ displayed a robust interaction with microorganisms. This led to an increase in current density in MES, reaching 2.5 times higher values compared to control MES setups lacking modified cathodes^33^. Similarly, an MES utilizing a porous framework gas diffusion electrode (GDE) exhibited enhanced current density, achieving 6 A/m^2^^34^. Although these porous materials have exhibited positive effects on current uptake in MESs, their constrained surface area (e.g., 278 m^2^/g in Fe_x_MnO_y_^33^ and 230–454 m^2^/g in GDE^35^) has hindered extensive biofilm development. Thus, to address this, the exploration of novel materials with larger surface areas becomes imperative. In this context, metal–organic frameworks (MOFs) present a promising alternative. Despite they have not been tested in MESs yet, these materials possess high physicochemical and electrochemical properties, rendering them highly viable as biocatalysts in MES applications. As an instance, MOFs exhibit an exceptional surface area of approximately 7000 m^2^/g^36^, accompanied by active ligands and metal nodes. Particularly, the Hong Kong University of Science and Technology Metal–Organic Framework (HKUST-1), is a versatile and well-studied MOF containing redox-active copper (Cu) sites coordinated with organic ligands. The presence of redox-active copper centers in HKUST in support of its impeccable porous matrix can potentially participate in electron transfer processes^2^. These attributes can effectively stimulate the development of electroactive biofilms^37^. However, to exploit the full potential of MOFs in MES, further modification in the matrix is required such as to improve the conductivity, stability, and affinity toward microbial attachment^38^. One such modification could be the hybridization of MOF with nitrogen-containing heterocycles like pyridinium ions to improve the biocatalytic feature of MOF^4,39^. The heterocyclic macromolecule g-C_3_N_4_ containing nitrogen can offer a great deal of catalytic activity as compared to other heterocycles due to their conductive feature and high surface area^40^.

The aim of this study is to understand the interaction of electroactive microbes with different MOF-based composite materials and the ability to perform extracellular electron exchange for CO_2_ reduction. The MOF (HKUST-1), *g-*C_3_N_4_, and *g-*C_3_N_4_-MOF composite were newly synthesized and assessed for their biocompatibility with microbes for biohybrid development. The developed biohybrids were tested using scanning electron microscopy (SEM), protein assimilation, and c-type cytochrome analysis, and the best material to develop biocathode over copper oxide (Cu_2_O|CuO) foam. The performance of biohybrid biocathodes for CO_2_ reduction to VFA was evaluated in single-chamber MESs and the results were compared with a control MES with abiotic Cu_2_O|CuO foam.

## Experimental

### Materials

Materials used in this research were of high quality such as BTC (1, 3, 5-benzene tri-carboxylic acid, 98%), Copper (II) nitrate (Cu(NO_3_)_2_·3H_2_O, 99%), Copper (II) sulfate (Cu(SO_4_)_2_·3H_2_O, 99%), phosphate buffer saline (PBS), NaHCO_3_, Cu plate, NaOH, H_2_SO_4_, melamine and PVDF (Polyvinyldifluoridone) as purchased from Merck. The solvent used in the preparation of materials was NMP (*N*-methyl pyrrolidone), DMF(*N*, *N*-dimethylformamide), and DI (deionized) water.

### ***Synthesis of g-C***_***3***_***N***_***4***_***-MOF composite***

The synthesis process of HKUST-1 was accomplished using a simple mixing procedure that included stirring and sonication with and without *g-*C_3_N_4_. A mixture of 1050.7 mg BTC and 639.99 mg NaOH were added in solvents such as DMF (20 ml), ethanol (150 ml), DI water (200 ml) and stirred vigorously to obtain a homogenous solution. In this liquid mixture, a 1208 mg of *g-*C_3_N_4_ (prepared by annealing recrystalline melamine at 550 °C for 4 h) was added and mixed by ultrasonication. Then, 50 ml water with 1208 mg of Cu(NO_3_)_2_ was added and mixed by ultrasonication. As prepared product *i.e.* *g-*C_3_N_4_-HKUST-1(MOF) particle was collected by centrifugation with consecutive washing by 30% ethanol and DI water. This composite wss named as *g-*C_3_N_4_-MOF. MOF and *g-*C_3_N_4_-MOF was prepared by mixing MOF or *g-*C_3_N_4_-MOF (36 mg) in 200 μl NMP containing 4 mg PVDF as a binder. The mixture was stirrered overnight to make a homogenous dispersion and fabricate electrode with smooth catalyst coating.

### Characterization of the prepared materials

Synthesized porous pristine and modified Cu foam films on Cu base material and powder materials were characterized using SEM (FEI Nova NanoSEM 450 outfitted with GENESIS software and an EDX detector). XRD patterns were collected using an X-ray diffractometer ( Bruker, D8 ADVANCE; scan speed = 4° min^–1^, Cu Kα radiation wavelength = 1.54060 Å). Fourier transform infrared (FTIR) spectroscopy measurements were carried out by a FTIR spectrometer (Nicolet iS10; scan rate of 2.5 cm s^–1^). X-ray photoelectron spectroscopy (XPS) observations were carried out using a Microlab 350 (Thermo Electron) spectrometer with a lateral resolution of 0.2 mm^2^ and Al-K non-monochromated radiation (1486.6 eV; 300 W) as the excitation source. The analysis was conducted at a pressure of 5.0 × 10^–9^ mbar. High-resolution and survey spectra were captured utilizing pass energies of 40 and 100 eV, respectively. Measurements of the electrochemical process were made using Palmsens potentiostat/galvanostat in a cell with a fixed at its base. An Ag/AgCl/KCl(3 M) and Pt wire were used as a counter and reference electrode, respectively on top of the working electrode. The electrolyte was 0.5 M phosphate buffer of pH 7.

### Fabrication of electrodes

A Cu plate of 0.5 mm thickness was used for making Cu foam. A current density of – 2 A/cm^2^ was applied in CuSO_4_·5H_2_O (0.2 M) in 1.5 m H_2_SO_4_ containing 20 mM NaCl for 20 s while keeping the Cu plate of the same dimension as a counter electrode. After the electrochemical process is completed, the Cu foam deposited on a Cu plate was cleaned with water flow and finally converted to Cu oxide foam (Cu_2_O|CuO) by annealing at 250 °C for 3 h. A 100 ml dispersion of *g-*C_3_N_4_-MOF (36 mg) was mounted on Cu_2_O|CuO surface and dried at 60 °C. The *g-*C_3_N_4_-MOF modified Cu_2_O|CuO were used for subsequent electrochemical charecterisation studies.

### ***g-C***_***3***_***N***_***4***_***-MOF-Bacteria biohybrid preparation and characterization***

The synthesized materials viz. *g-*C_3_N_4_-MOF, *g-*C_3_N_4_, MOF, were dispersed in 25 ml fresh water medium (FWM) with the following ingredients: 2.5 g/L NaHCO_3_, 1.75 g/L NaH_2_PO_4_, 4.6 g/L Na_2_HPO_4_, 0.165 g/L KNO_3_, 1 ml/L Vitamine solution and 5 ml/L Mineral solution in a 100 mL culture bottle^37^. The dispersion was sonicated at 10 kHz ultrasound frequency for 30 min to obtain a homogenous mixture. The solution was deaerated using a CO_2_/H_2_ (70:30) gas mixture for 15 min (10 min inside the solution and 5 min in the headspace) to make the solution fully anaerobic and then the rubber stoppers were sealed using aluminum caps. The culture bottles were autoclaved at 120 °C for 30 min. After the bottles were cooled down, the mixture was sonicated one more time at 5 kHz ultrasound frequency for 5 min. The microbial seed as inoculum was obtained from a running MES, which mainly contained electroautotrophic microbes such as *Proteobacteria, Bacteroidetes, and Firmicutes*^41^. Briefly, the suspended microbial cells obtained from the cathode chamber of an MES were collected and centrifuged (3000 rpm, 10 min) to separate the granular materials and metabolites. The microbial cell suspension was further washed a couple of times using a zwitterionic sulfonic acid buffering agent (HEPES, Sigma Aldrich, the USA) and adjusted to the OD ~ 0.8. The harvested cells were then inoculated in the culture bottles @ 20% V/V. The culture bottles were incubated in a shaker incubator at 30 °C (100 RPM) and purged with CO_2_/H_2_ every 24 h. After every 15 days, the *g-*C_3_N_4_-MOF-microbial biohybrid (CMB), *g-*C_3_N_4_-microbial biohybrid (CB) MOF-microbial biohybrid (MB), and control P (planktonic cells only) was recovered using a centrifuge, washed with HEPES, and reshuffled in a new FWM. At least four reshufflings (~ 60 days) were done to allow the bacteria to grow inside the materials.

The microbial population in the biohybrids was determined using protein analysis using a protein assay kit (Pierce™ BCA Protein Assay Kits, Thermo Fisher Scientific, US). Further, cytochrome quantification per unit microbial protein was conducted using a cytochrome detection kit (Abcam, US)^42^. The surface morphology of biohybrids was analyzed using SEM to understand the interaction of the microbes with the materials. In brief, the biohybrid granules were dehydrated using a series of diluted ethanol (10–90%) followed by fixing the microbes using a 2% glutaraldehyde solution^43,44^. The specimens were then carefully placed on the silicon wafers and coated with platinum for SEM analysis.

### MES set-up and operation

Selected biohybrids (CMB and MB) at a loading rate of 5 mg/cm^2^ (with 30 μl/ml Nafion 117 solution) were applied to the 1 cm^2^ Cu_2_O|CuO plate using the drop-casting method in anaerobic conditions. The electrodes were kept in a sealed container having CO_2_/H_2_ for 24 h. The electrodes were tested for microbe-assisted CO_2_ reduction in a single-chamber MES. Two MESs viz. with CMB|Cu_2_O|CuO (MES-1), MB|Cu_2_O|CuO (MES-2), and a control MES-3 (only with *g-*C_3_N_4_-MOF|Cu_2_O|CuO) were made with glass material (50 total volume) having a lid with a rubber gasket. The lid had the provision for inserting an anode (4 cm^2^ titanium wire mesh), an Ag/AgCl reference electrode, gas purging, and sample collection ports. The electrolyte contained FWM with 4 g NaHCO_3_ (as the sole soluble carbon source) and was purged with argon gas before starting the reaction. The MES was operated with − 0.8 V applied voltage (*vs* Ag/AgCl) under chronoamperometric mode for three days. The experiments were conducted in a homemade portable globe box filled with argon gas to minimize the intrusion of oxygen. The experiments were repeated at least three times to validate the authenticity of the data.

### Analysis and calculations

The liquid sample obtained from the MESs was analyzed in ion chromatography (IC, Metroohm, the USA) to measure the VFA production. The IC machine had an organic acid ion exchanger column (Metrosep Organic Acids—250/7.8, USA) that could measure a wide range of VFA in the water and wastewater samples. The liquid samples obtained from MESs were filtered using 0.2 μm syringe filters and diluted properly to bring the VFA concentrations within the calibration range (~ 10 mM for each VFA component). A ~ 0.5 ml sample was injected into the IC column. The VFA components were eluted using 10 mM LiCl and 0.25 mM H_2_SO_4_ mobile phase at a flow rate of 1 ml/min and pressure of ~ 8.5 Mpa. The chromatograms were fitted in the 3-point calibration curve to measure the actual concentration of different VFA. The coulombic efficiency (CE) was calculated using the following Eq. (1)^45^.

1 $${\text{CE}} = \frac{{F \times \sum \left( {X_{i} \times n_{i} } \right)}}{{\mathop \smallint \nolimits_{to}^{t} Idt}}$$

where *F* is the Faraday constant (96,485 C/mol), *Xi* is the amount of constituent of VFA in mol/L (*i* is individual VFAs), *n* is the number of electrons required for conversion of individual VFA, and *I* is the current response recorded during chronoamperometry.

## Results and discussion

### Physiochemical characterization of composite materials

**Figure 1:**
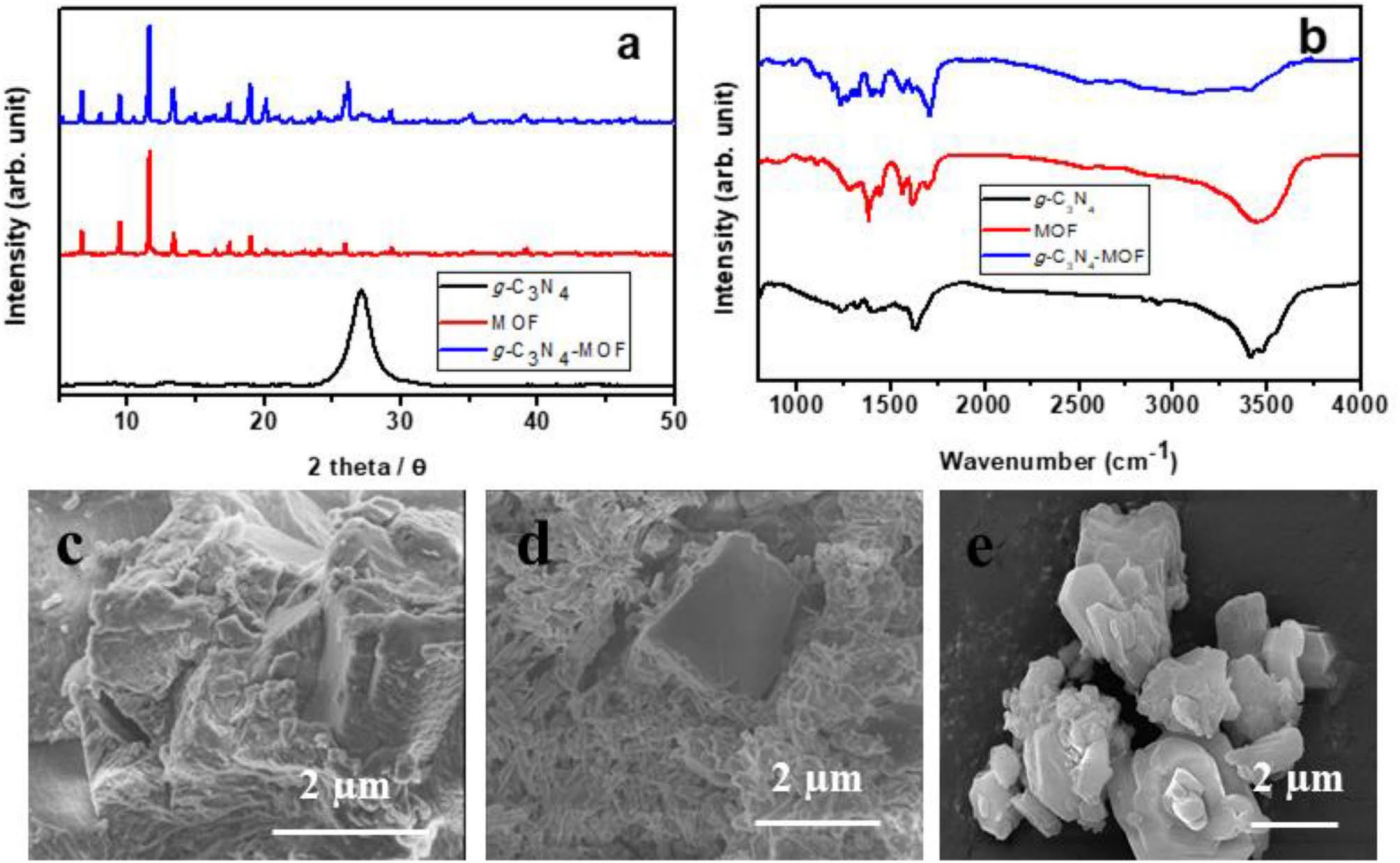
Physiochemical characterization of materials. (**a**) FT-IR (**b**) XRD pattern and (**c**–**e**) The SEM images of the powder sample of *g-*C_3_N_4_, MOF, and g-C_3_N_4_-MOF.

As synthesized materials were comprehensively characterized to evaluate their structural, surface, morphological, and electrochemical characterization. Such characterization can justify their suitability to use in MESs for CO_2_ reduction. Initially, the materials were characterized by FT-IR and X-ray diffraction (XRD) patterns to identify the interaction and immobilization of *g-*C_3_N_4_ with MOF as shown in Fig. 1a,b.

XRD of MOF illustrates its crystalline nature and after the incorporation of *g-*C_3_N_4_ crystallinity remains intact (Fig. 1a). Slight appearance of *g-*C_3_N_4_ peak at 20.6° confirms its presence in the MOF matrix^46^. The BTC linker's vibrations are represented by the FT-IR peaks below 1200 cm^−1^(Fig. 1b). The coordination of BTC to the copper sites is what causes the bands between 1300 and 1700 cm^−1^ to belong to the carboxylate linker. The asymmetric stretching vibrations of the carboxylate in BTC are responsible for the peak at 1646 cm^−1^. The carboxylate groups' symmetric stretching vibrations are related to the peaks at 1448 and 1371 cm^−1^. The CN aromatic repeating unit of *g-*C_3_N_4_ such as C–N and C=N stretching vibrations were responsible for the many absorption peaks in the range of 1200–1640 cm^−1^. All the peaks belonging to MOF show a slight shift in the high wavenumber side due to charge transfer interaction with *g-*C_3_N_4_. Peaks belonging to *g-*C_3_N_4_ appeared at the same position with weak intensity due to its small amount in the matrix. The SEM analysis also confirms *g-*C_3_N_4_-MOF sample preparation in the form of particles of ∼ 5–10 μm in size and octahedral in shape similar to the previous literature^47^ as shown in Fig. 1c–e. However, immobilization of *g-*C_3_N_4_ does not change the morphology of MOF.

**Figure 2:**
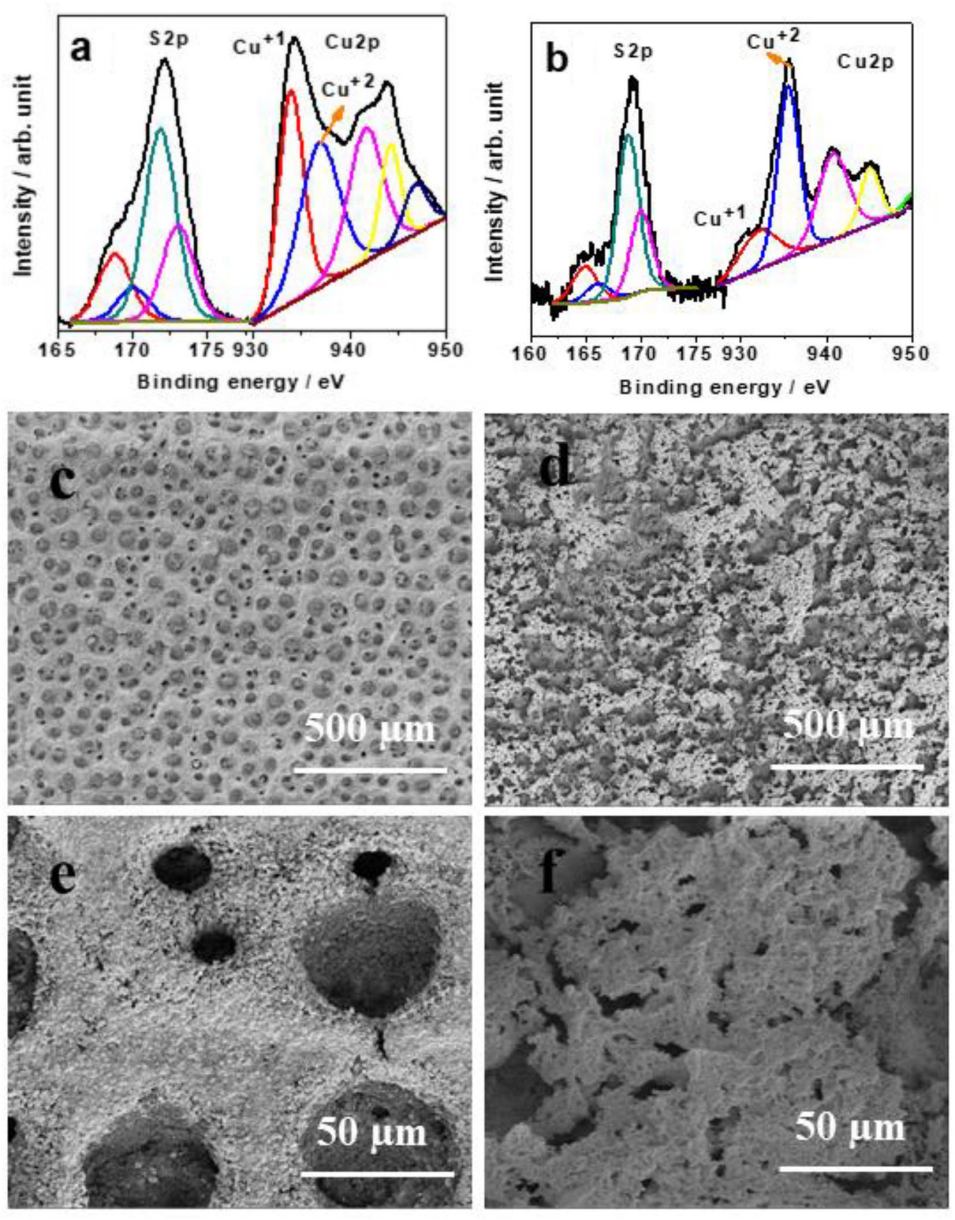
High-resolution XPS scan spectra of Cu 2p and S 2p of (**a**) Cu_2_O/CuO foam and (**b**) *g-*C_3_N_4_-MOF immobilized in Cu_2_O/CuO foam. The SEM images of (**c**–**e**) Cu_2_O/CuO foam and (**d**–**f**) *g-*C_3_N_4_-MOF immobilized in Cu_2_O/CuO foam from low to high magnification.

XPS was used to examine the chemical constitution of the electrocatalysts present on the Cu_2_O|CuO foam substrate without microorganisms and compare it to pristine Cu_2_O|CuO foam. According to Fig. 2a, the peaks at 933.2 eV are Cu2p_3/2_ of the Cu2p spectrum, which are related to the synthesis of Cu^+1^^48^. Additional distinct peaks at 936.5 eV that can be attributed to Cu2p_3/2_ phases of Cu^2+^ show the presence of very little CuO on the uppermost layer. After the deposition of *g-*C_3_N_4_-MOF on Cu_2_O|CuO foam Cu^+2^ peak enhances as a symptom of MOF with Cu^+1^ along with carbon (C–N and C–C) and sulfur feature (Fig. 2b). Other than that both peaks shifted to the low binding energy side as a result strong electron-donating effect of BTC in comparison to an oxygen atom. The existence of the Cu^+1^ feature even after *g-*C_3_N_4_-MOF deposition supports its impregnation into the pore of Cu_2_O|CuO foam instead of complete surface coverage.

The Cu_2_O|CuO foam was used as a support system for the fabrication of electrodes. The SEM image of the Cu_2_O|CuO foam clearly shows uniformly distributed pores (Fig. 2c and e), which are a key requirement for microbes to attach with the electrodes.

Further, the impregnation of *g-*C_3_N_4_-MOF in the Cu_2_O|CuO pore was examined. As a result of impregnation, the micrometer size of the pore, which is seen in the case of Cu_2_O|CuO foam, disappeared with the filling of *g-*C_3_N_4_-MOF (Fig. 2d and f). Moreover, a network like structure with a small size pore comes out as a substitute for the micrometer size of a pore of Cu_2_O|CuO foam.

### Electrochemical characterization of electrodes under abiotic and biotic conditions

**Figure 3:**
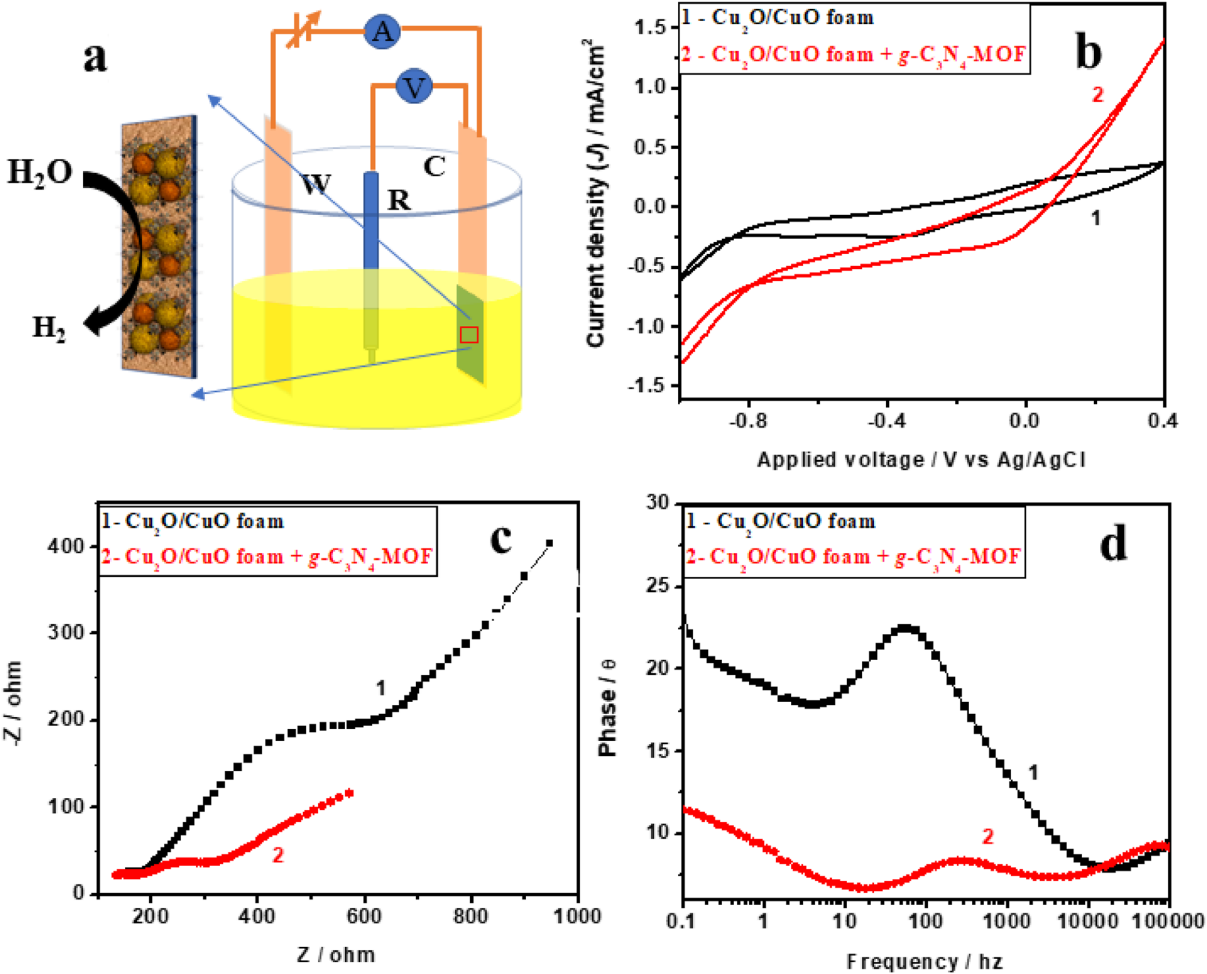
(**a**) Schematic illustration of electrochemical characterization of Cu_2_O|CuO foam modified with *g-*C_3_N_4_-MOF (**b**) cyclic voltammogram (**c**) Nyquist plots (**d**) Bode plots of Cu_2_O|CuO foam and Cu_2_O|CuO foam modified with *g-*C_3_N_4_-MOF in 0.5 M phosphate buffer saline.

The prepared electrodes *i.e.* Cu_2_O|CuO foam and Cu_2_O|CuO foam modified with *g-*C_3_N_4_-MOF were evaluated for their electrocatalytic activity to understand their possibility as support for microbial growth on the electrode (Fig. 3a–d). The electrocatalytic activities of electrodes with electrocatalysis were studied using CO_2_-saturated 0.5 M phosphate buffer saline (PBS) electrolyte in a three electrode electrochemical cell (Fig. 3a). The fresh Cu_2_O|CuO foam was also tested as control in the same scenario to compare the results. The CV curves of the samples are shown in Fig. 3b.

Cathodic peaks of Cu_2_O|CuO foam can be seen naturally before water reduction, which could be attributed to the reduction of Cu^+2^ to Cu. The optimized Cu_2_O|CuO with *g-*C_3_N_4_-MOF sample requires a comparatively low overpotential of -750 mV for H_2_ evolution compared to pure Cu_2_O|CuO, which needs − 810 mV to achieve current densities of 1 mA/cm^2^. Catalytic current at − 800 mV potential is approximately double in the case of Cu_2_O|CuO with *g-*C_3_N_4_-MOF sample to that of Cu_2_O|CuO, which is crucial for electrocatalytic reduction^49^. The electrochemical surface areas (ECSA) of electrocatalysts can be measured from the double-layer capacitance (C_dl_) in the impedance spectrum.

The obtained impedance spectrum was modeled in Nyquist and Bode algorithm and further used to better understand the HER kinetics process (Fig. 3c). The low-frequency arc in Nyquist plot is related to the resistance caused by the adsorption of reaction intermediates at the electrode surface, whereas the high-frequency arc is connected to the interfacial charge transfer process (R_ct_)^50^. The graph clearly shows that Cu_2_O|CuO with *g-*C_3_N_4_-MOF electrodes exhibit much smaller charge transfer resistances (523 Ohm) than that (1616 Ohm) of blank Cu_2_O|CuO with *g-*C_3_N_4_-MOF, indicating that more facile charge transport occurs on Cu_2_O|CuO with *g-*C_3_N_4_-MOF electrodes during HER process.

Bode plot (Fig. 3d) further indicates the high phase angle (23°) probably because of low charge transfer resistance and high surface area as a result of significant double layer capacitive contribution.

**Figure 4:**
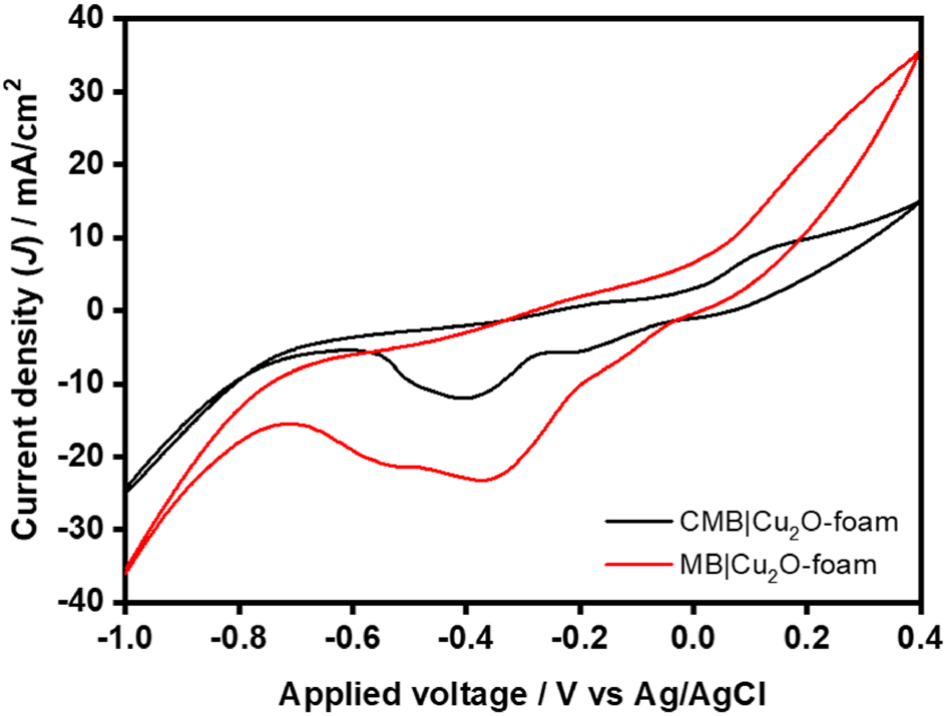
Cyclic voltammetry analysis of biocathodes.

Furthermore, the electrochemical properties of the fabricated biohybrid cathodes viz*. g-*C_3_N_4_-MOF biohybrid (CMB) and MOF biohybrids (MB) i.e., CMB|Cu_2_O|CuO and MB|Cu_2_O|CuO, electrode were analyzed using CV in the applied potential window of + 400 mV to − 1.0 V (vs Ag/AgCl) at a scan rate of 10 mV/s (Fig. 4). The voltammogram showed a significant increase in the reduction current from 1.4 mA/cm^2^ to 23.2 mA/cm^2^ in CMB|Cu_2_O|CuO and 1.1 mA to 11.7 in MB at the potential window starting from ~ 0 mV to − 400 mV. For this reason, the rise in the cathodic current can be attributed to the CO_2_ reduction to acetate and butyrate. A similar rise in the cathodic current was also observed in the case of microbial biocathodes^51,52^. A sharp rise in the cathodic current was further noticed after − 700 mV in both the biocathodes, which can be attributed to the hydron evolution reaction (HER)^53^. The CMB|Cu_2_O|CuO demonstrates a significantly high HER current of 36.4 mA as compared to MB|Cu_2_O|CuO (24 mA). Both the cathodes also demonstrated redox current peaks at different positions, for instance, CMB|Cu_2_O|CuO showed two pairs of redox peaks at − 500 mV and − 230 mV and − 350 mV and 230 mV, whereas MB|Cu_2_O|CuO demonstrated the redox peaks at − 390 mV and − 180 mV and − 190 mV and − 120 mV, respectively. These redox peaks can be attributed to the reduction and oxidation of electron shuttles in microbes during the extracellular electron exchange processes. For instance, Shoparwe *et. al.* observed a couple of redox peaks reduction peaks at 170 mV and − 700 mV (vs. Saturated Calomel Electrode, SCE) with complementary oxidation peaks at − 600 mV and 500 mV (vs. SCE) for *Geobacter*-enriched biofilm^54^. Moreover, the current exchange during both the oxidation and reduction cycle for CMB|Cu_2_O|CuO was noted higher as compared to the MB|Cu_2_O|CuO cathode, indicating that the CMB|Cu_2_O|CuO was a more active biocatalyst as compared to MB|Cu_2_O|CuO. This result corroborates the earlier VFA production results during biohybrid preparation and MES tests.

### Biohybrid development and assessment

Initially, the utilized materials (viz *g-*C_3_N_4_-MOF, *g-*C_3_N_4_, and MOF) and the planktonic cell after inoculation were fully dispersed in the solution (Fig. S2) and after a week of culture, small microbial granules started forming in the solution (all there culture bottles except for the culture with only planktonic cell), signifying that the microbes were getting attached with the materials as shown in the FESEM micrograph (Fig. S2). The protein concentration in the biohybrids viz. CMB (*g-*C_3_N_4_-MOF-bacteria), CB (*g-*C_3_N_4_-bacteria), and MB (MOF-bacteria) were measured at the end of the final shuffling (after 60 days). The CMB had a higher protein concentration of 28.6 ± 3.5 mg/g-CMB, whereas the CB and MB showed protein assimilation of 18.3 ± 1.5 mg/g-CB and 24.3 ± 3.5 mg/g-MB. Moreover, the c-type cytochromes concentration was estimated in the biohybrid granules as well as the planktonic microbes. Interestingly, the c-type cytochrome concentration was also noted to be high in CMB (0.05 ± 0.003 unit/mg-protein) as compared to the CB (0.035 ± 0.005 units/mg-Protein) and MB (0.044 units/mg-Protein). Whereas, the planktonic cells only had 0.011 ± 0.002 units/m-Protein. This means that *g-*C_3_N_4_-MOF favors an excellent attachment of microbes under autotrophic conditions as compared to only *g-*C_3_N_4_ and MOF. There could be several possible explanations for this result and the most prominent reason among them could be the excellent structural feature of the *g-*C_3_N_4_-MOF. Particularly, MOF is a highly porous matrix with a tuned surface area, which facilitated a large surface to the microbes and also helped pre-concentrate the CO_2_/H_2_ inside the matrix^55,56^. Moreover, the utilization of *g-*C_3_N_4_ inside the MOF matrix greatly elevated the conductivity of the material, which assisted with low-resistance electron–electron transfer.

VFA production in the culture bottles with CMB, CB, MB, P, and dead-cell control (DC) was monitored daily. All four culture systems, except DC, could reduce CO_2_ into representable acetic acid and a like amount of iso-butyric acid during each shuffle. The VFA production in all the culture systems was noted to be gradually increased until the end of the shuffling time (Fig. S3) and reached 153 ± 12 mg/L and 66 ± 8 mg/L, respectively for CMB, 114 ± 8 mg/L and 21 ± 4 mg/L, respectively for CB, 144 ± 6 mg/L and 36 ± 3 mg/L, respectively for MB and 90 ± 5 mg/L and 15 ± 2 mg/L for P. However, the average equivalent chemical oxygen demand (COD) yield was noted significantly higher in CMB followed by MB, CB, and P (Fig. S3). By contrast, the DC did not show any VFA generation, demonstrating that the cathode electrode without bacteria is not capable of overcoming the CO_2_ reduction overpotential barrier. Based on the above results, the CMB and MB were chosen for further testing in MES as biocatalysts for the cathode.

### Performance analysis of Microbial electrosynthesis using CMB and MB cathodes

#### Current uptake

**Figure 5:**
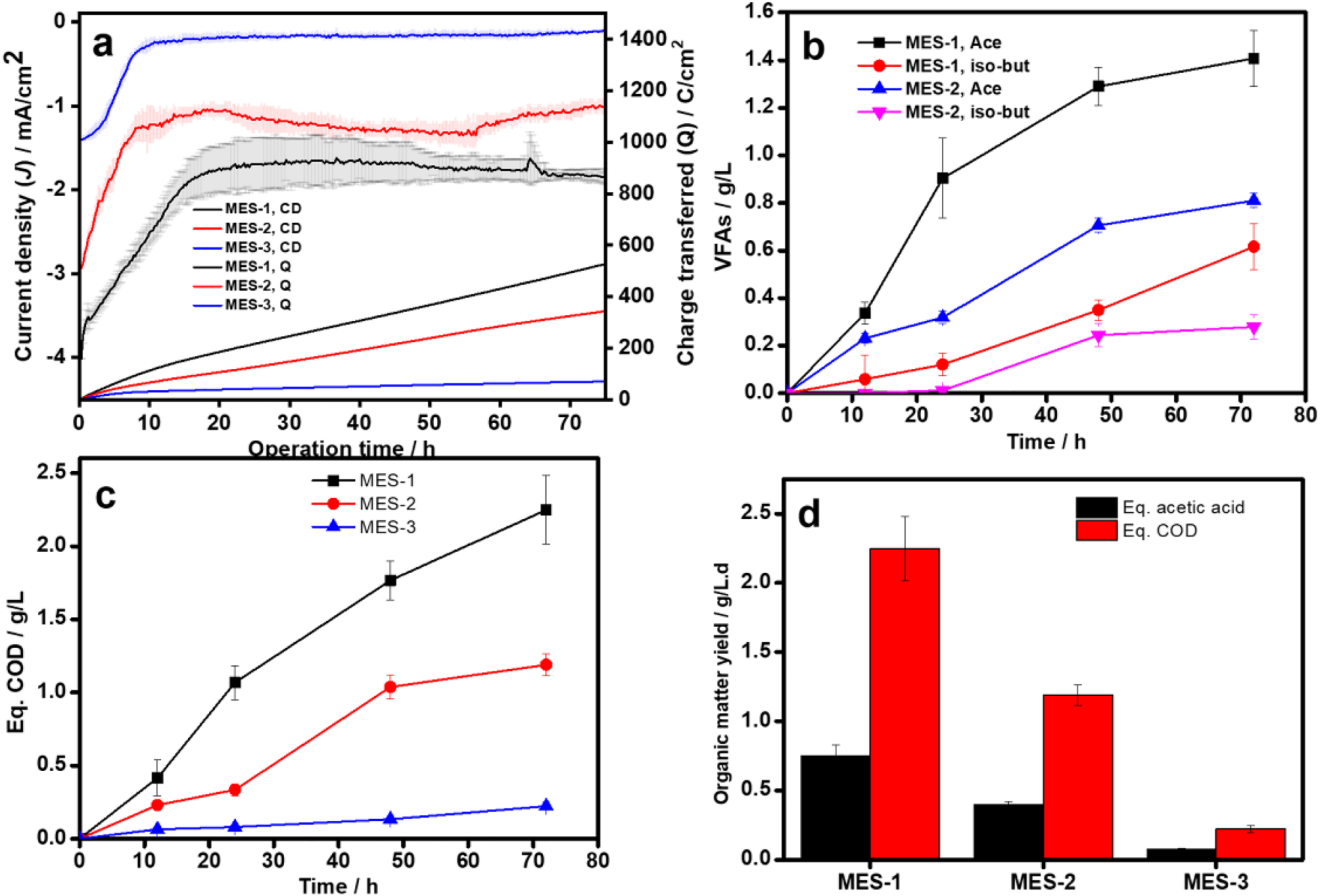
Performance of MESs using different biohybrid materials (**a**) Current response and corresponding charge uptake by microbes (**b**) VFA production (**c**) Equivalent COD production and (**d**) Acetic acid and COD yield.

The MES with two different biohybrid cathodes viz. CMB|Cu_2_O|CuO (MES-1), and MB| Cu_2_O|CuO (MES-2) were operated using chronoamperometry at a fixed applied potential of − 0.8 V (vs. Ag/AgCl). The results of both the MESs were compared with a control MES-3 (with abiotic *g-*C_3_N_4_-MOF|Cu_2_O|CuO). All the MES showed a initial decrease in the negative current and then remained constant for 3 days (Fig. 5a). This decrease in the current can be attributed to the charge balance by counter-positive charges (K^+^, Na^+^, H^+^) available in the electrolyte^57^. After balancing the positive charges, the current response became constant followed by electron consumption for CO_2_ reduction in the MESs. However, the MES-1 had a significantly higher current response with stable average current production of 1.7 ± 0.06 mA/cm^2^ as compared to the MES-2 (1.1 ± 0.1 mA/cm^2^) within three days of operation. MES-2 could also produce higher current due to enhanced catalytic activity of the cathode as compared to the recent studies, for instance, 0.076 mA/cm^2^ in MES with the plain electrode and *Desulfovibrio G11* strain biohybrid^58^, 0.15 mA/cm^2^ in MES using a plain electrode and *IS4 microbial* strain biohybrid^59^ and 1 mA/cm^2^ in MES using NiMo-biohybrid catalysed cathode^60^. By contrast, the MES-3 could only produce an average stable current density of 0.14 ± 0.2 mA/cm^2^ throughout the operation period. This signifies that the microbes contributed to a large portion of current consumption through their autotrophic metabolic pathways in the MES-1 and MES-2. Thus, the biotic cathode is superior as compared to abiotic cathode for the electroreduction of CO_2_. However, the MES-1 had a higher current uptake than that of the MES-2 possibly due to a high microbial population density in the biohybrid (as shown by protein and c-type cytochrome concentration). The total charge consumption was also higher in MES-1 with a value of 505 C as compared to the MES-2 (327 C) and significantly higher than that of the MES-3 (65 C), which could be utilized by the microbes for CO_2_ reduction to VFAs (Fig. 5a).

#### Volatile fatty acid production

The VFA production in the MESs was monitored every 24 h. MES-1 and MES-2 could reduce CO_2_ into acetic acid and a small amount iso-butyric acid, whereas samples taken from control MES-3 contained only formic acid with a little amount of acetic acid. Usually, the CO_2_ reduction on abiotic electrocatalyst surfaces produces mostly C1 organic acids (*i.e.*, formic acid) due to the limited ability of chain elongation reaction in inorganic catalysts^61^. However, the microbes are not limited by this feature and they can perform chain elongation to produce higher chain fatty acids such as acetatic, butyric, valeric acids, etc. The bioelectrochemical transformation of acetatic and butyric acids in MES occurs through a multistep CO_2_ reduction pathway^62^. In the first step, CO_2_ reduces to Acetyl-Coenzyme A (CoA)—a central metabolite—through the Wood–Ljungdahl pathway, and then Acetyl-CoA undergoes phosphorylation to yield acetate. Whereas acetyl-CoA undergoes a series of enzymatic reactions such as yielding acetoacetyl-CoA and butyryl-CoA, leading synthesis of butyrate via reverse β-oxidation^63^. Therefore, microbial CO_2_ reduction could be a better alternative to obtain more valuable medium-chain fatty acids for industrial applications. The VFA production in terms of acetic and iso-butyric acids in MES-1 and MES-2 was noted to increase with the increase in the reaction time (Fig. 5b). The highest acetic and iso-butyric acid production after 3 days was noted to be 1.4 ± 0.1 g/L and 0.6 ± 0.09 g/L, respectively for MES-1 and 0.8 ± 0.03 g/L and 0.27 ± 0.05 g/L, respectively for MES-2. On the other hand, the MES-3 could only produce 0.18 ± 0.02 g/L formic acid and a small amount of acetic acid ~ 0.02 g/L after 3 days of reaction time. The equivalent COD in all three MES was also noted to be increased with days of operation and reached to the concentration of 2.2 ± 0.2 g/L, 1.2 ± 0.1 g/L, and 0.2 ± 0.02 g/L, respectively, after 3 days of operation (Fig. 5c). The equivalent COD concentration also suggests MES-1 much superior among the tested MESs. In terms of equivalent acetatic acid production rate (Fig. 5d), the MES-1 also had superiority with an average yield of 0.72 ± 0.07 g/L.d among the recently reported values (Table 1). The results suggest that g-C_3_N_4_-MOF (HKUST-1) composite is an excellent biocompatible material to integrate electroactive microbes for MES applications. Moreover, the composite contained a highly porous surface with active ligands and metal nodes such as carboxylic acid and Cu.

**Table 1:** Performance comparison of irreverent MESs.

MES type	Cathode materials	Microbe	Imposed potential/V vs. Ag/AgCl*	Products	Acetate production, g/L.d	CE, %	Refs.
Dual chamber	Chitosan on carbon cloth	*S. Ovata*	− 0.4	Acetate	0.32	86	^68^
Dual chamber	3D Iron oxide carbon felt	*S. Ovata*	− 0.69	Acetate	0.24	86	^69^
Dual chamber	Mo_2_C-CF	Mixed culture	− 0.85	Acetate	0.19	64	^70^
Dual-chamber	Graphene–nickel foam	Mixed cultures	− 0.85	Acetate	0.013	70	^71^
Single-chamber	Fe_x_MnO_y_	Mixed culture	− 0.8	Acetate and iso-butyrate	0.38	58	^33^
Single-chamber	*g-*C_3_N_4_-MOF biohybrid	Mixed culture	− 0.8	Acetate and iso-butyrate	0.75	95	This study

**Figure 6:**
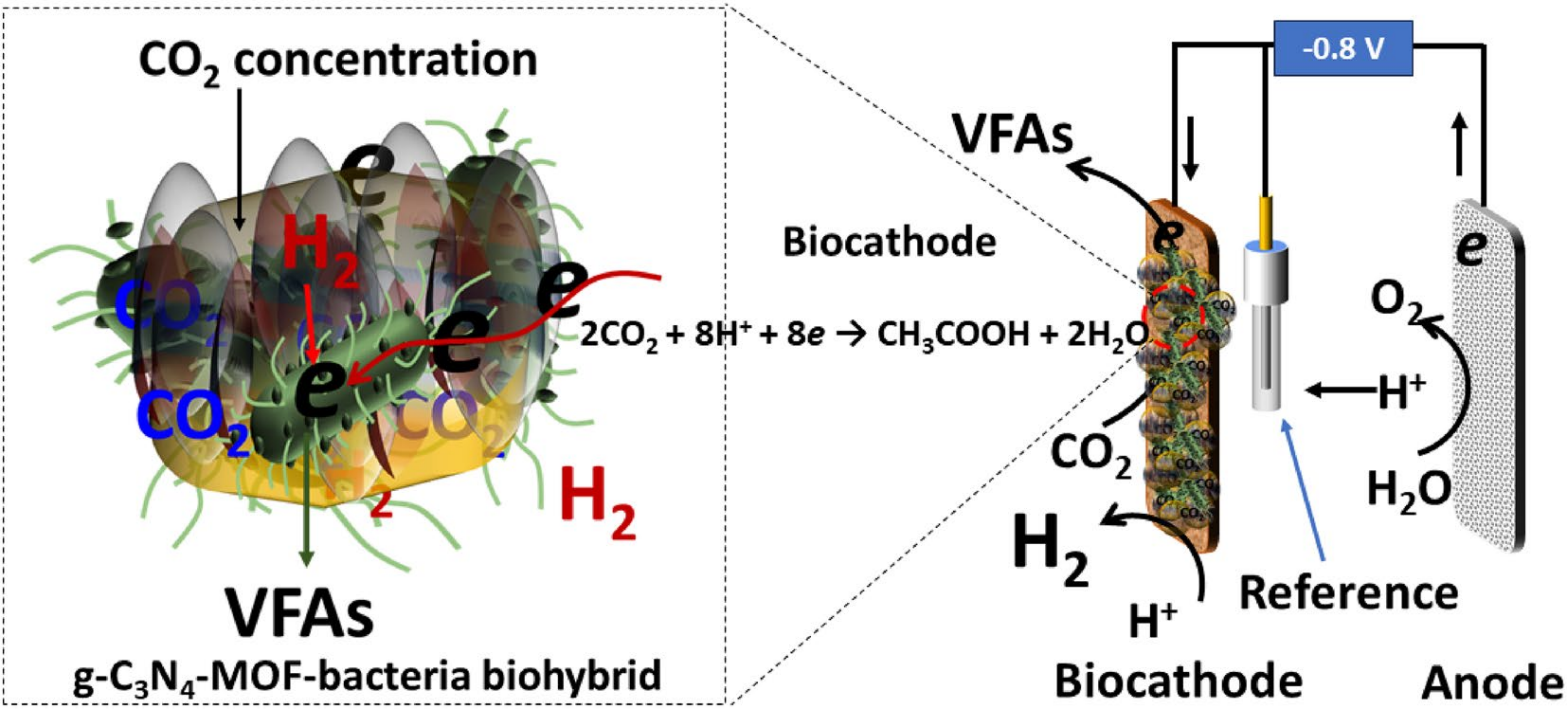
Schematic illustration of mechanism of volatile fatty acids formation through microbial electrosynthesis.

This imparts multiple benefits such as providing an additional surface area for the microbes to form colonies and substrate preconcentration in the vicinity of the microbes, ultimately reducing the concentration overpotential losses (Fig. 6). Additionally, *g-*C_3_N_4_ has good electrical conductivity with high electron mobility, which supports extracellular electron transfer from the cathode to the microbes, thus improving the current uptake by the microbes^64^.

Therefore, the MES with CMB|Cu_2_O|CuO could demonstrate high VFA production as compared to the other MESs tested in this study as well as the recent studies as shown in Table 1. However, the MOFs, in particular, are organic frameworks, and thus, they may undergo a slow degradation process, decreasing their stability and long-term applications in MESs^65^. Moreover, after degradation, the metal nodes in the MOFs can contaminate the environment. Therefore, it is necessary to further investigate the impact of MOFs on the environment before their widespread applications in both abiotic and biotic applications. The headspace gas composition in all three MESs was also monitored over the reaction period (Fig. S3). After 3 days of the experimental period, the MES-1 had a hydrogen accumulation of 0.89 ± 0.08 mM, which was noted significantly higher than that of the MES-2 (0.66 ± 0.04 mM) and control MES-3 (0.47 ± 0.06 mM).

The hydrogen accumulation in the MES is a result of the HER contributed by the autotrophic microbes and in the case of MES-1, the CMB biocatalyst accelerated the HER. Similarly, in other studies as well, the HER was also noted to be accelerated by the biocathodes developed on the Cu-foam substrate^66^ and other biometals^67^. The overall coulomb conversion ratio was also noted to be high in MES-1 (95 ± 9%) as compared to MES-2 (78 ± 5%), MES-3 (73 ± 8%), and other recent studies (Table 1). Overall results indicate that the CMB is an efficient biocatalyst for the bioelectrochemical reduction of CO_2_ to medium-chain fatty acids at a high rate.

## Conclusion

This study demonstrates the development of an efficient biohybrid biocatalyst for bioelectrochemical CO_2_ reduction in MES. The *g-*C_3_N_4_-MOF (HKUST-1) provided an excellent support for the microbes to develop electroactive biohybrid catalyst and transport electrons on the matrix as compared to pristine *g-*C_3_N_4_ and MOF. As a result, the MES with CMB mobilized on Cu_2_O|CuO-foam base cathode yielded significantly high acetatic acid of 0.72 g/L.d with a CE of 95%, as compared to MES with MB|Cu_2_O|CuO and control (without any biohybrid). Thus, development of electroactive biohybrids using HKUST-1 framework could be an excellent strategy to develop biocathode for application in scaled-up MESs.

### Supplementary Information

Supplementary Figures.

## Data Availability

The datasets used and/or analyzed during the current study are available from the corresponding author upon reasonable request.

## References

[1] Choi YH, Jang YJ, Park H, Kim WY, Lee YH, Choi SH, Lee JS (2017). Carbon dioxide Fischer-Tropsch synthesis: A new path to carbon-neutral fuels. Appl. Catal. B Environ..

[2] Wang D, Xu J, Zhu Y, Wen L, Ye J, Shen Y, Zeng T, Lu X, Ma J, Wang L (2021). HKUST-1-derived highly ordered Cu nanosheets with enriched edge sites, stepped (211) surfaces and (200) facets for effective electrochemical CO_2_ reduction. Chemosphere.

[3] Tayyebi E, Hussain J, Abghoui Y, Skúlason E (2018). Trends of electrochemical CO_2_ reduction reaction on transition metal oxide catalysts. J. Phys. Chem. C..

[4] Elouarzaki K, Kannan V, Jose V, Sabharwal HS, Lee J (2019). Recent trends, benchmarking, and challenges of electrochemical reduction of CO_2_ by molecular catalysts. Adv. Energy Mater..

[5] Albo J, Beobide G, Castaño P, Irabien A (2017). Methanol electrosynthesis from CO_2_ at Cu_2_O/ZnO prompted by pyridine-based aqueous solutions. J. CO2 Util..

[6] Roy S, Cherevotan A, Peter SC (2018). Thermochemical CO_2_ hydrogenation to single carbon products: Scientific and technological challenges. ACS Energy Lett..

[7] Yu J, Low J, Xiao W, Zhou P, Jaroniec M (2014). Enhanced photocatalytic CO_2_-reduction activity of anatase TiO_2_ by coexposed 001 and 101 facets. J. Am. Chem. Soc..

[8] Ahmad N, Anae J, Khan MZ, Sabir S, Yang XJ, Thakur VK, Campo P, Coulon F (2021). Visible light-conducting polymer nanocomposites as efficient photocatalysts for the treatment of organic pollutants in wastewater. J. Environ. Manag..

[9] Kong Q, Kim D, Liu C, Yu Y, Su Y, Li Y, Yang P (2016). Directed assembly of nanoparticle catalysts on nanowire photoelectrodes for photoelectrochemical CO_2_ reduction. Nano Lett..

[10] Kuhl KP, Cave ER, Abram DN, Jaramillo TF (2012). New insights into the electrochemical reduction of carbon dioxide on metallic copper surfaces. Energy Environ. Sci..

[11] Zhang S, Kang P, Meyer TJ (2014). Nanostructured tin catalysts for selective electrochemical reduction of carbon dioxide to formate. J. Am. Chem. Soc..

[12] Loiudice A, Lobaccaro P, Kamali EA, Thao T, Huang BH, Ager JW, Buonsanti R (2016). Tailoring copper nanocrystals towards C2 products in electrochemical CO_2_ reduction. Angew. Chemie Int. Ed..

[13] Yoo JS, Christensen R, Vegge T, Nørskov JK, Studt F (2016). Theoretical insight into the trends that guide the electrochemical reduction of carbon dioxide to formic acid. ChemSusChem.

[14] Hori Y, Takahashi I, Koga O, Hoshi N (2003). Electrochemical reduction of carbon dioxide at various series of copper single crystal electrodes. J. Mol. Catal. A Chem..

[15] Geioushy RA, Khaled MM, Hakeem AS, Alhooshani K, Basheer C (2017). High efficiency graphene/Cu_2_O electrode for the electrochemical reduction of carbon dioxide to ethanol. J. Electroanal. Chem..

[16] Lim RJ, Xie M, Sk MA, Lee J-M, Fisher A, Wang X, Lim KH (2014). A review on the electrochemical reduction of CO_2_ in fuel cells, metal electrodes and molecular catalysts. Catal. Today.

[17] Ganesh I (2014). Conversion of carbon dioxide into methanol–a potential liquid fuel: Fundamental challenges and opportunities (a review). Renew. Sustain. Energy Rev..

[18] Nguyen HTT, Noori MT, Min B (2022). Accelerating anaerobic digestion process with novel single chamber microbial electrochemical systems with baffle. Bioresour. Technol..

[19] Noori MT, Min B (2022). Fundamentals and recent progress in bioelectrochemical system assisted biohythane production. Bioresour. Technol..

[20] Nitopi S, Bertheussen E, Scott SB, Liu X, Engstfeld AK, Horch S, Seger B, Stephens IEL, Chan K, Hahn C (2019). Progress and perspectives of electrochemical CO_2_ reduction on copper in aqueous electrolyte. Chem. Rev..

[21] Senocrate A, Battaglia C (2021). Electrochemical CO_2_ reduction at room temperature: Status and perspectives. J. Energy Storage.

[22] Li D, Zhang H, Xiang H, Rasul S, Fontmorin J-M, Izadi P, Roldan A, Taylor R, Feng Y, Banerji L (2021). How to go beyond C 1 products with electrochemical reduction of CO_2_. Sustain. Energy Fuels.

[23] Noori MT, Mohan SV, Min B (2021). Microbial electrosynthesis of multi-carbon volatile fatty acids under the influence of different imposed potentials. Sustain. Energy Technol. Assess..

[24] Li X, Angelidaki I, Zhang Y (2018). Salinity-gradient energy driven microbial electrosynthesis of value-added chemicals from CO_2_ reduction. Water Res..

[25] Marshall CW, Ross DE, Fichot EB, Norman RS, May HD (2013). Long-term operation of microbial electrosynthesis systems improves acetate production by autotrophic microbiomes. Environ. Sci. Technol..

[26] Bajracharya S, Yuliasni R, Vanbroekhoven K, Buisman CJN, Strik DPBT, Pant BD (2017). Long-term operation of microbial electrosynthesis cell reducing CO_2_ to multi-carbon chemicals with a mixed culture avoiding methanogenesis. Bioelectrochemistry.

[27] Li, D., Noori, M. T., Ng, K. S., Liu, G. & Yu, E. H. The development of cathode materials for boosting CO_2_ conversion in microbial electrosynthesis cells. In *Material Microbes Interactions Environmental Biotechnological Perspective* Ch. 7, 171–198 (2023).

[28] Anwer AH, Khan N, Khan MD, Shakeel S, Khan MZ (2021). Redox mediators as cathode catalyst to boost the microbial electro-synthesis of biofuel product from carbon dioxide. Fuel.

[29] Ali RB, Noori MT, Lee S-H, Park H-D, Min B (2022). Enhancing biogas and electricity recovery using an iron-manganese oxide catalyzed bioanode in an integrated submersible microbial fuel cell-anaerobic digester. Sustain. Energy Technol. Assess..

[30] Noori MT, Thatikayala D, Pant D, Min B (2021). A critical review on microbe-electrode interactions towards heavy metal ion detection using microbial fuel cell technology. Bioresour. Technol..

[31] Noori MT, Vu MT, Ali RB, Min B (2020). Recent advances in cathode materials and configurations for upgrading methane in bioelectrochemical systems integrated with anaerobic digestion. Chem. Eng. J..

[32] Park S-G, Rajesh PP, Sim Y-U, Jadhav DA, Noori MT, Kim D-H, Al-Qaradawi SY, Yang E, Jang J-K, Chae K-J (2022). Addressing scale-up challenges and enhancement in performance of hydrogen-producing microbial electrolysis cell through electrode modifications. Energy Rep..

[33] Noori MT, Min B (2019). Highly porous FexMnOy microsphere as an efficient cathode catalyst for microbial electrosynthesis of volatile fatty acids from CO_2_. ChemElectroChem.

[34] Bajracharya S, Vanbroekhoven K, Buisman CJN, Pant D, Strik DP (2016). Application of gas diffusion biocathode in microbial electrosynthesis from carbon dioxide. Environ. Sci. Pollut. Res..

[35] Srikanth S, Pant D, Dominguez-Benetton X, Genné I, Vanbroekhoven K, Vermeiren P, Alvarez-Gallego Y (2016). Gas diffusion electrodes manufactured by casting evaluation as air cathodes for microbial fuel cells (MFC). Materials.

[36] Flexer V, Jourdin L (2020). Purposely designed hierarchical porous electrodes for high rate microbial electrosynthesis of acetate from carbon dioxide. Acc. Chem. Res..

[37] Wang C, Ye X, Liu Y, Jia Z, Cao C, Xiao Q, Du J, Kong X, Wu X, Chen Z (2022). Enhanced anaerobic digestion for degradation of swine wastewater through a Fe/Ni-MOF modified microbial electrolysis cell. J. Clean. Prod..

[38] Shakeel S, Khan MZ (2022). Enhanced production and utilization of biosynthesized acetate using a packed-fluidized bed cathode based MES system. J. Environ. Chem. Eng..

[39] Ehrmaier J, Rabe EJ, Pristash SR, Corp KL, Schlenker CW, Sobolewski AL, Domcke W (2019). Singlet–triplet inversion in heptazine and in polymeric carbon nitrides. J. Phys. Chem. A..

[40] Gupta P, Verma N (2022). Conversion of CO_2_ to formate using activated carbon fiber-supported *g-*C_3_N_4_–NiCoWO_4_ photoanode in a microbial electrosynthesis system. Chem. Eng. J..

[41] Lust R, Nerut J, Gadegaonkar SS, Kasak K, Espenberg M, Visnapuu T, Mander Ü (2022). Single-chamber microbial electrosynthesis reactor for nitrate reduction from waters with a low-electron donors’ concentration: From design and set-up to the optimal operating potential. Front. Environ. Sci..

[42] Noori MT, Ghangrekar MM, Mukherjee CK (2016). V_2_O_5_ microflower decorated cathode for enhancing power generation in air-cathode microbial fuel cell treating fish market wastewater. Int. J. Hydrog. Energy.

[43] Vu MT, Noori MT, Min B (2020). Conductive magnetite nanoparticles trigger syntrophic methane production in single chamber microbial electrochemical systems. Bioresour. Technol..

[44] Vu MT, Noori MT, Min B (2020). Magnetite/zeolite nanocomposite-modified cathode for enhancing methane generation in microbial electrochemical systems. Chem. Eng. J..

[45] Mohanakrishna G, Vanbroekhoven K, Pant D (2018). Impact of dissolved carbon dioxide concentration on the process parameters during its conversion to acetate through microbial electrosynthesis. React. Chem. Eng..

[46] Fina F, Callear SK, Carins GM, Irvine JTS (2015). Structural investigation of graphitic carbon nitride via XRD and neutron diffraction. Chem. Mater..

[47] Lin R, Ge L, Diao H, Rudolph V, Zhu Z (2016). Ionic liquids as the MOFs/polymer interfacial binder for efficient membrane separation. ACS Appl. Mater. Interfaces.

[48] Roy A, Jadhav HS, Seo JG (2021). Cu_2_O/CuO electrocatalyst for electrochemical reduction of carbon dioxide to methanol. Electroanalysis.

[49] Wu Y, Li Y, Lü Z, Xu L, Wei B (2020). Heterostructural Ni_3_S_2_–Fe_5_Ni_4_S_8_ hybrids for efficient electrocatalytic oxygen evolution. J. Mater. Sci..

[50] Das I, Noori MT, Bhowmick GD, Ghangrekar MM (2018). Application of low-cost transition metal based Co_0.5_Zn_0.5_Fe_2_O_4_ as oxygen reduction reaction catalyst for improving performance of microbial fuel cell. MRS Adv..

[51] Rajesh PP, Noori MT, Ghangrekar MM (2018). Graphene oxide/polytetrafluoroethylene composite anode and chaetoceros pre-treated anodic inoculum enhancing performance of microbial fuel cell. J. Clean Energy Technol..

[52] Choi O, Kim T, Woo HM, Um Y (2014). Electricity-driven metabolic shift through direct electron uptake by electroactive heterotroph Clostridium pasteurianum. Sci. Rep..

[53] Wang Y, Li M, Ren H (2022). Voltammetric mapping of hydrogen evolution reaction on Pt locally via scanning electrochemical cell microscopy. ACS Meas. Sci. Au.

[54] Shoparwe, N. F., Makhtar, M. M. Z., Sata, S. A., Kew, W.S., Mohamad, M. & Shukor, H. Cyclic voltammetry studies of bioanode microbial fuel fells from batch culture of Geobacter sulfurreducens. In *IOP Conference Series: Earth and Environmental Science* 12102 (IOP Publishing, 2021).

[55] Thatikayala D, Noori MT, Min B (2023). Zeolite-modified electrodes for electrochemical sensing of heavy metal ions–Progress and future directions. Mater. Today Chem..

[56] Noori MT, Ezugwu CI, Wang Y, Min B (2022). Robust bimetallic metal-organic framework cathode catalyst to boost oxygen reduction reaction in microbial fuel cell. J. Power Sources.

[57] Harris AR, Newbold C, Carter P, Cowan R, Wallace GG (2018). Charge injection from chronoamperometry of platinum electrodes for bionic devices. J. Electrochem. Soc..

[58] Croese E, Pereira MA, Euverink G-JW, Stams AJM, Geelhoed JS (2011). Analysis of the microbial community of the biocathode of a hydrogen-producing microbial electrolysis cell. Appl. Microbiol. Biotechnol..

[59] Deutzmann JS, Spormann AM (2017). Enhanced microbial electrosynthesis by using defined co-cultures. ISME J..

[60] Kracke F, Wong AB, Maegaard K, Deutzmann JS, Hubert MKA, Hahn C, Jaramillo TF, Spormann AM (2019). Robust and biocompatible catalysts for efficient hydrogen-driven microbial electrosynthesis. Commun. Chem..

[61] Gupta P, Noori MT, Núñez AE, Verma N (2021). An insight into the bioelectrochemical photoreduction of CO_2_ to value-added chemicals. iScience.

[62] Jiang Y, Zeng RJ (2018). Expanding the product spectrum of value added chemicals in microbial electrosynthesis through integrated process design—A review. Bioresour. Technol..

[63] Lee H-S, Xin W, Katakojwala R, Mohan SV, Tabish NMD (2022). Microbial electrolysis cells for the production of biohydrogen in dark fermentation—A review. Bioresour. Technol..

[64] Tan X, Kou L, Tahini HA, Smith SC (2015). Conductive graphitic carbon nitride as an ideal material for electrocatalytically switchable CO_2_ capture. Sci. Rep..

[65] Singh N, Qutub S, Khashab NM (2021). Biocompatibility and biodegradability of metal organic frameworks for biomedical applications. J. Mater. Chem. B.

[66] Aryal N, Wan L, Overgaard MH, Stoot AC, Chen Y, Tremblay PL, Zhang T (2019). Increased carbon dioxide reduction to acetate in a microbial electrosynthesis reactor with a reduced graphene oxide-coated copper foam composite cathode. Bioelectrochemistry.

[67] Tremblay P-L, Angenent LT, Zhang T (2017). Extracellular electron uptake: Among autotrophs and mediated by surfaces. Trends Biotechnol..

[68] Zhang T, Nie H, Bain TS, Lu H, Cui M, Snoeyenbos-West OL, Franks AE, Nevin KP, Russell TP, Lovley DR (2013). Improved cathode materials for microbial electrosynthesis. Energy Environ. Sci..

[69] Cui M, Nie H, Zhang T, Lovley D, Russell TP (2017). Three-dimensional hierarchical metal oxide–carbon electrode materials for highly efficient microbial electrosynthesis. Sustain. Energy Fuels.

[70] Tian S, Wang H, Dong Z, Yang Y, Yuan H, Huang Q, Song TS, Xie J (2019). Mo_2_C-induced hydrogen production enhances microbial electrosynthesis of acetate from CO_2_ reduction. Biotechnol. Biofuels.

[71] Song T, Fei K, Zhang H, Yuan H, Yang Y, Ouyang P, Xie J (2018). High efficiency microbial electrosynthesis of acetate from carbon dioxide using a novel graphene–nickel foam as cathode. J. Chem. Technol. Biotechnol..

