# Supplementary material for: Copper foam supported g-C3N4-metal–organic framework bacteria biohybrid cathode catalyst for CO2 reduction in microbial electrosynthesis
Source: Sci Rep. 2023 Dec 20;13:22741. doi: 10.1038/s41598-023-49246-3 (PMC10733401; doi:10.1038/s41598-023-49246-3)

**Copper foam supported g-C3N4-metal-organic framework bacteria biohybrid cathode catalyst for CO2 reduction in microbial electrosynthesis**

Md Tabish Noori^a^, Mansi^b^, Shashank Sundriyal^c^, Vishal Shrivastav^d^, Balendu Sekhar Giri^e^, Marcin Holdynski^d^, Wojciech Nogala^d^, Umesh K. Tiwari^b^, Bhavana Gupta^d*^, Booki Min^a*^

^a^Department of Environmental Science and Engineering, Kyung Hee University, Yongin, South Korea;

^b^CSIR-Central Scientific Instrument Organisation (CSIR-CSIO), Chandigarh 160030, India;

^c^Regional Center of Advanced Technologies and MaterialsThe Czech Advanced Technology and Research Institute (CATRIN) Palacký University OlomoucŠlechtitel ̊u 27, Olomouc 779 00, Czech Republic;

^d^Institute of Physical Chemistry Polish Academy of Sciences, Kasprzaka 44/52, 01-224 Warszawa, Poland;

^e^Sustainability Cluster, School of Advanced Engineering, UPES, Dehradun, Uttarakhand, 248007, India

Corresponding authors: bgupta1206@gmail.com, bgupta@ichf.edu.pl (B. Gupta) and bkmin@khu.ac.kr (Booki Min)

Supplementary

Figure S1: g-C3N4-MOF biohybrid (a) Biohybrid development in a controlled environment (a, b, c) FESEM micrograph of CMB at different magnifications


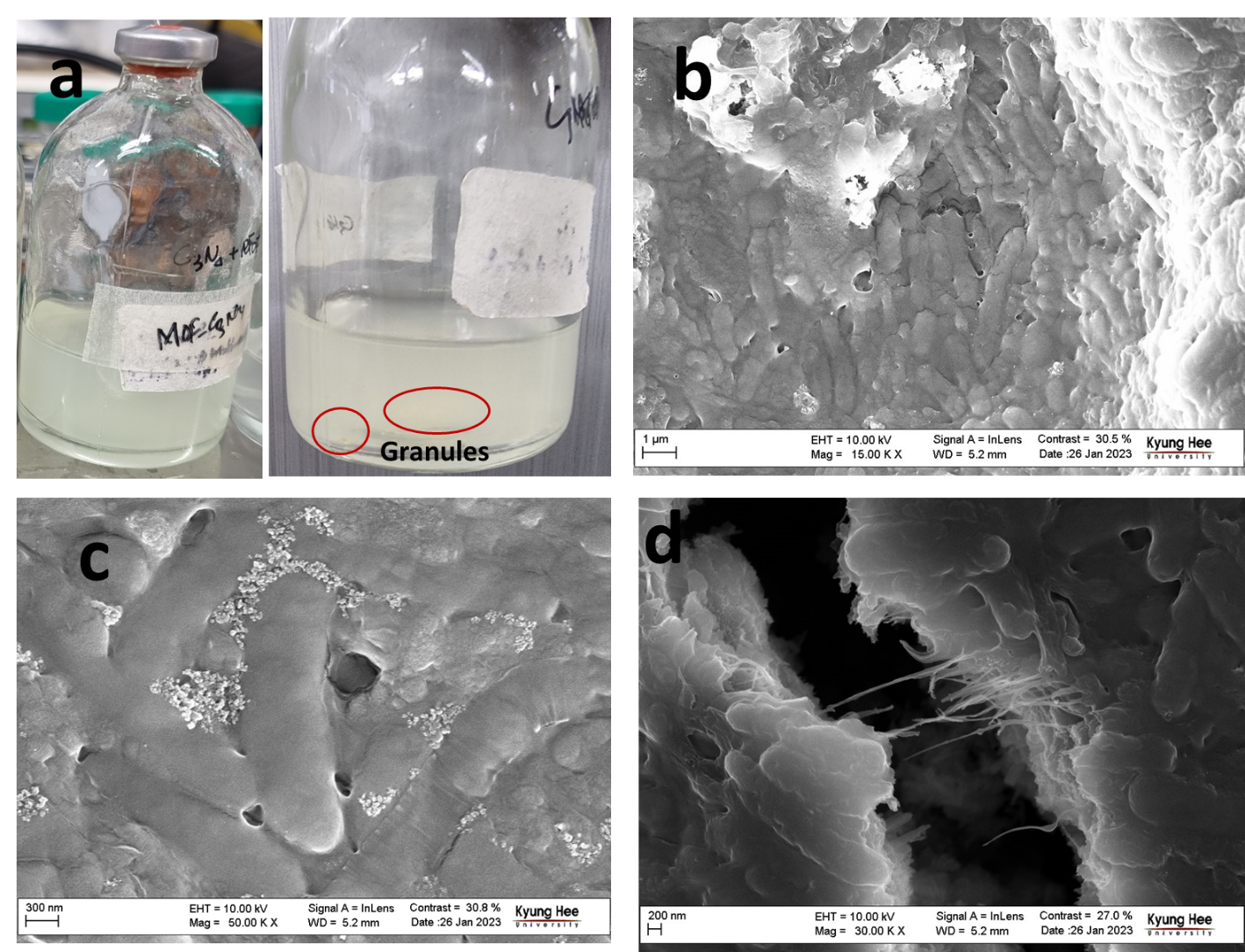


Figure S2: Organic acid production during biohybrid preparation (a) VFA concentration profile (b) Equivalent COD concentrations at the end of the experiment.


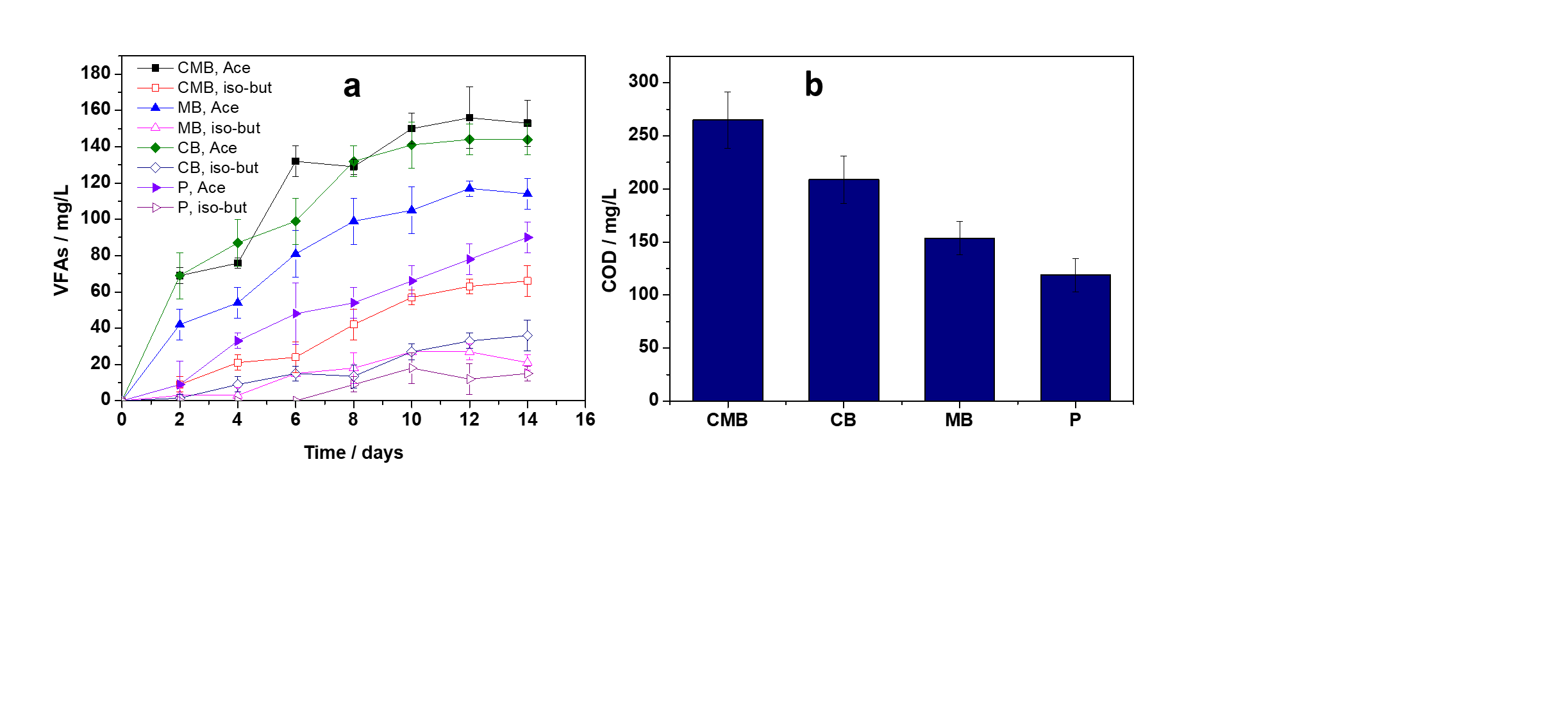


Figure S3: Head gas compositions


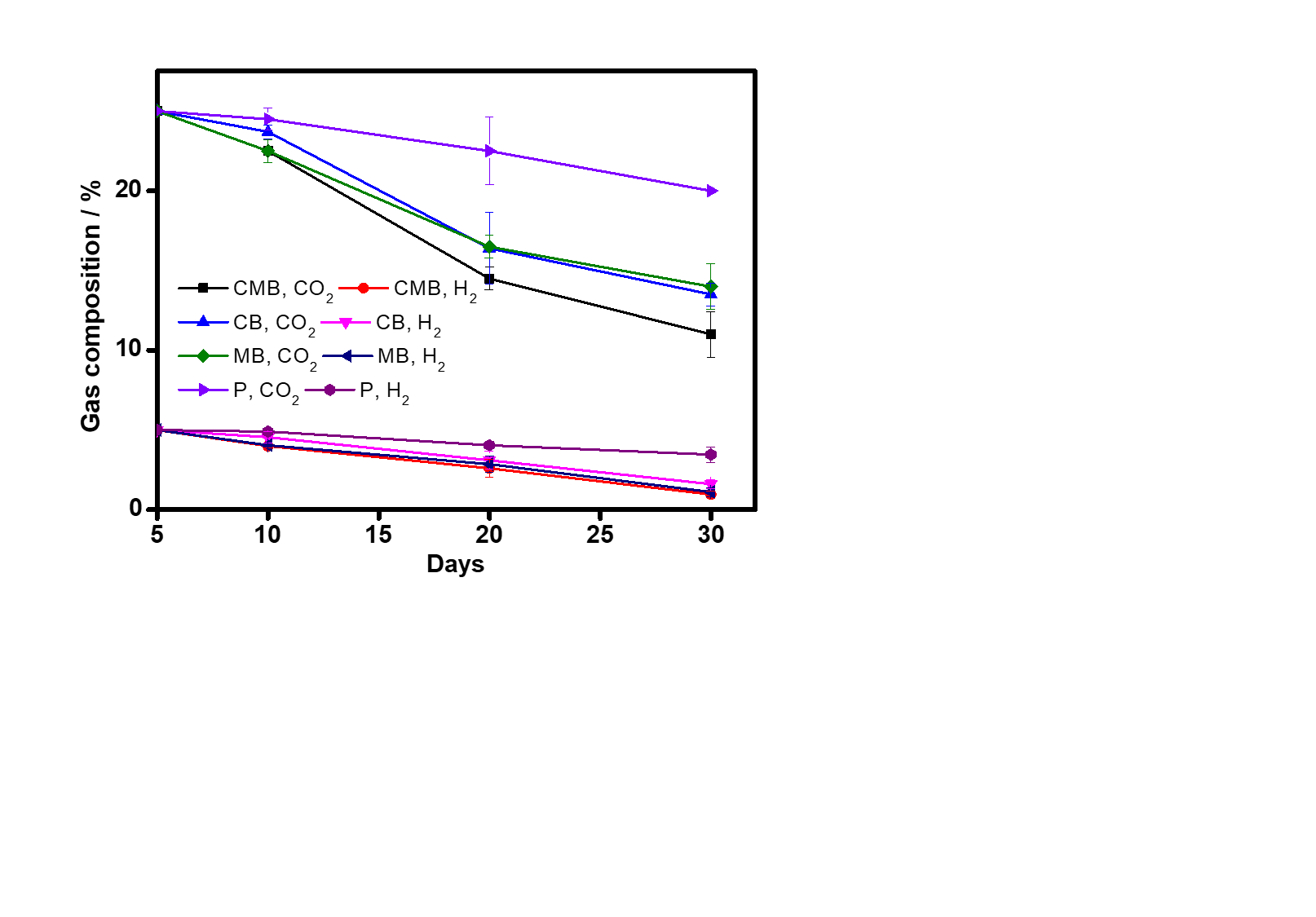

Supplement: Supplementary file 1 — Supplementary Figures. [file 41598_2023_49246_MOESM1_ESM.docx]
